# Relationship between *MEG3* gene polymorphism and risk of gastric cancer in Chinese population with high incidence of gastric cancer

**DOI:** 10.1042/BSR20200305

**Published:** 2020-11-24

**Authors:** Xiaoling Kong, Sheng Yang, Caiping Liu, Hanqing Tang, Yingan Chen, Xiaomei Zhang, Yun Zhou, Geyu Liang

**Affiliations:** 1 Key Laboratory of Environmental Medicine Engineering, Ministry of Education, School of Public Health, Southeast University, Nanjing, Jiangsu 210009, P.R. China; 2 Department of Medicine, Jiangsu Cancer Hospital and Jiangsu Institute of Cancer Research, Nanjing Medical University Affiliated Cancer Hospital, Nanjing, Jiangsu 210009, P.R. China

**Keywords:** environmental risk factors, gastric cancer, MEG3, risk assessment model, single nucleotide polymorphism

## Abstract

**Objective:** Gastric cancer is the most common gastrointestinal malignancy in China and results from a combination of genetic and environmental factors. The present study was conducted to investigate the relationship between long noncoding RNA (lncRNA) materally expressed gene 3 (*MEG3*) single nucleotide polymorphisms (SNPs) and the risk of gastric cancer and to construct a genetic-environmental risk assessment model. **Methods:** A case–control study was conducted to include 474 patients with gastric cancer diagnosed by clinical and pathological examination and 543 healthy physical examination subjects. Blood samples, general demographic data and behavioral lifestyle of the subjects were collected. The TaqMan real-time PCR method was used for testing the genotypes of *MEG3* rs7158663 and rs10132552. **Results:** The A allele at the rs7158663 loci of *MEG3* was found to be risk factor for gastric cancer (odds ratio (OR) = 1.41, 95% confidence interval (95% CI) = 1.14–1.74, *P*=0.002). Yet, no significant association between rs10132552 polymorphisms and gastric cancer was observed. Drinking, tea drinking and preserved food eating were risk factors for gastric cancer (*P*<0.05). A genetic–environmental risk assessment model was established by using the logistic regression model to include *MEG3* rs7158663, drinking, tea drinking, and preserved food eating. With the increase in risk score (RS), the risk of gastric cancer increased substantially (*P*<0.05). And the area under the receiver operating characteristic (ROC) curve was 0.745, which indicates a high diagnostic value. **Conclusions:** *MEG3* rs7158663 might be associated with the risk of gastric cancer; the diagnostic ability of genetic–environmental risk assessment model for gastric cancer is better.

## Introduction

As one of the malignant tumors with high morbidity and mortality, gastric cancer is a serious threat to human health. According to the latest cancer statistics, there are ∼27510 new cases of gastric cancer in the United States in 2019, and ∼11140 people died from the disease [1]. In China, gastric cancer is the most common malignant tumor of the digestive tract and its morbidity and mortality rate rank second among all malignant tumors [2]. In Wuwei, Gansu Province, the incidence of gastric cancer is high, and the mortality rate is as high as 90.71/100000, ranking first in China [3]. The eating habits of the region are like hot and sour, long-term consumption of smoked and preserved food. High levels of carcinogens such as nitrites and polycyclic aromatic hydrocarbons in food are important causes of high incidence of gastric cancer in this area. At present, there are few studies on the risk factors of Wuwei gastric cancer. Mi et al. [4] showed that the genetic susceptibility factor accounted for approximately one-fifth to one-fourth in the formation of gastric cancer familial aggregation. However, the occurrence of gastric cancer is mainly the result of the interaction of multiple factors such as genetic factors, environmental, and social factors. Therefore, it is important to study the risk of gastric cancer from environmental and genetic levels.

With the deepening research, it is found that not all individuals will develop gastric cancer when exposed to the same environmental risk factors (ERFs), suggesting that there are differences in genetic susceptibility between individuals [5]. As one of the most common forms of genomic variation, single nucleotide polymorphism (SNP) refers to a DNA sequence polymorphism caused by a single nucleotide variation at the genomic level [6]. The study of SNP is helpful to explain the genetic and pathological mechanisms of complex diseases and the interaction between genes and the environment [7,8], so that we can better target high-risk groups and perform early screening, diagnosis and treatment.

Current studies on the relationship between genetic polymorphisms and gastric cancer susceptibility have been reported. He et al. [9] demonstrated that ERCC1 rs2298881 CA variant genotype was associated with an increased gastric cancer risk, while ERCC1 rs3212986 AA variant genotype was identified as a protective factor for gastric cancer. Hua et al. [10] found that genetic variations in LIG3 gene may play a weak role in modifying the risk of gastric cancer. Long noncoding RNA (lncRNA) refers to a transcript with more than 200 nucleotides, usually not encoding a protein, but has multiple functions [11]. As one of cancer-related lncRNAs, materally expressed gene 3 (*MEG3*) is a human homolog of the imprinted gene *Glt2* of the mouse mother which was first discovered by Miyoshi et al. in 2000, as a type of imprinted gene mapped to human chromosome 14q32.3 [12]. *MEG3* is normally expressed in many tissues in the human body, such as placenta, adrenal gland, brain and pituitary gland [13], but is reduced in tumor cells such as gastric cancer [14], liver cancer, and lung cancer [12]. Genomic association studies have found that SNP on lncRNA is associated with the development of many cancers, such as lung cancer [15] and prostate cancer [16]. Studies on the relationship between *MEG3* SNP and tumor susceptibility have also been reported. Related research showed that that subjects carrying *MEG3* rs4081134 AG/AA genotypes significantly tended to develop neuroblastoma among subgroups with age > 18 months, when compared with reference group [17].

According to our previous research results, the expression of *MEG3* is decreased in gastric cancer tissues [18]. On this basis, *MEG3* rs7158663 and rs10132552 were selected through preliminary literature research [19–21]. Candidate SNPs genotyping were performed by TaqMan probe-fluorescence PCR method and the relationship between candidate SNPs and the risk of gastric cancer was discussed. Furthermore, combining the general demographic data and behavioral lifestyle information of the research subjects, we attempt to establish a genetic–environmental risk assessment model of gastric cancer, which provides a reference for the prevention and diagnosis of gastric cancer in high-risk areas in China.

## Materials and methods

### Population samples

From January 2016 to July 2017, peripheral blood of 474 patients with gastric cancer was collected from the Wuwei Tumor Hospital of Gansu Province as a case group, while 543 peripheral blood samples from normal people were collected as healthy controls. These patients were diagnosed with gastric cancer by histopathology at Wuwei Tumor Hospital, Gansu Province. The blood was collected under aseptic conditions, placed in an EDTA anticoagulation tube, and stored at −80°C. Besides, information on general demographics, behavioral lifestyles, and family history of cancer was also collected. These subjects consented in advance and signed informed consent forms and the study was approved by the Medical Ethics Committee of Wuwei Tumor Hospital. The response rate for cases and controls was over 90%.

### SNPs selection and genotyping

In the Chinese Han population, the NCBI dbSNP database was used to select potential SNPs with minor allele frequencies greater than 20% reported in HapMap, which were rs7158663 and rs10132552, respectively. The extracted genomic DNA was amplified by TaqMan real-time PCR, and the PCR amplification primers for rs7158663 and rs10132552 SNPs were shown in Table 1. The reaction conditions were set as follows: 40 cycles at 95°C for 10 min, 95°C for 15 s, annealing at 60°C for 1 min, and finally at 60°C for 30 s. Loading Sterilization of deionized water instead of DNA as template was used as the negative control. Five percent of the samples were randomly selected for repeated experiments, and the consistency of the repeated samples was 100%, which indicated that the genotyping results are reliable.

**Table 1:** Primer sequence of MEG3 SNPs

SNPs	Primer sequence
MEG3 rs7158663	F: GGGATGCTGAGATTCGGGATA
	R: GACCTTGTGGGTCTGGTACAGAA
MEG3 rs10132552	F: GAAACCAACATCCCACATACTCTAAC
	R: TCTCTTTGTCCCTCCCCAGTT

‘F’ refers to forward primer; ‘R’ refers to reverse primer.

### Construction of genetic–environmental risk assessment model

A genetic–environmental risk assessment model was established using a weighted method, with integrated genetic risk factors (GRFs) and ERFs to comprehensively assess the overall risk of gastric cancer. The score of GRF was calculated as the sum of risk alleles of each SNP (0, 1, or 2 copies per risk allele). ERFs are categorical variables, such as smoking were classified as smokers and nonsmokers, with values of 1 and 0, respectively. The risk score (RS) was constructed based on the combination of genetic and ERFs weighted by their coefficients (β), which were calculated by logistic regression analysis. The specific formula is as follows: RS = β_1_* GRF + β_2_ * ERF [22].

In order to ensure prediction accuracy, samples were divided into four groups according to the RS: 0 (Q < 25), 1 (Q25–Q50), 2 (Q50–Q75), and 3 (≥Q75). The lowest group was used as the reference group. Moreover, the diagnostic ability of the genetic-environmental risk assessment model was evaluated using the area under the curve (AUC) of the receiver operating characteristic (ROC) curve.

### Statistical analysis

The genotype and allele frequencies of the case and the control groups were calculated using SPSS 23.0 statistical software (SPSS, Chicago, IL, U.S.A.). The χ^2^ test was used to verify whether the genotype distribution of each SNP conforms to the Hardy–Weinberg genetic equilibrium law. The association between polymorphism and risk of gastric cancer was analyzed by logistic regression analysis. The odds ratio (OR) and 95% confidence interval (95% CI) were used to estimate the relationship between each variable and the risk of gastric cancer. Continuous variables were expressed as 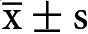, and *t* test was used for comparison between groups. *P*<0.05 indicated that the difference was statistically significant.

## Results

### Basic characteristics of samples

Characteristics of case and control samples are shown in Table 2. Among 474 patients with gastric cancer, 266 (56.12%) were males and 208 (43.88%) were females, with an average age of 58.00 ± 6.98 years. Among 543 controls, 283 (52.12%) were males and 260 (47.88%) were females, with an average age of 57.41 ± 5.49 years. There were no significant differences in distribution of gender and age between the case and the control groups (*P*>0.05). Alcohol drinking, tea drinking, and preserved food eating were significantly associated with the incidence of gastric cancer (*P*<0.05).

**Table 2:** Relationship among demographics, environmental factors, and risk of gastric cancer

Characteristics	Cases (*n*=474)	Controls (*n*=543)	*P-*value
Gender (*n* (%))			0.202
Female	208 (43.88)	260 (47.88)	
Male	266 (56.12)	283 (52.12)	
Age (years, mean ± SD)	58.00 ± 6.98	57.41 ± 5.49	0.136
Drinking (*n* (%))			**<0.001**
No	336 (70.89)	446 (82.14)	
Yes	138 (29.11)	97 (17.86)	
Tea drinking (*n* (%))			**<0.001**
No	455 (95.99)	386 (71.09)	
Yes	19 (4.01)	157 (28.91)	
Eating pickled food (*n* (%))			**<0.001**
No	54 (11.39)	164 (30.20)	
Yes	420 (88.61)	379 (69.80)	
Family history of cancer (*n* (%))			0.086
No	426 (89.87)	469 (86.37)	
Yes	48 (10.13)	74 (13.63)	

Note: Bold values mean that the difference in the distribution of research factors between the case and the control group is statistically significant.

### *MEG3* polymorphisms and the susceptibility of gastric cancer

In the case group, rs7158663 GG accounted for 45.36%, GA accounted for 41.77%, AA type accounted for 12.87%, and in the control group they accounted for 53.41, 37.38, and 9.21%, respectively. The results showed that in the codominant model, GA genotype carriers had a 42% increased risk of gastric cancer compared with the GG genotype (OR = 1.42, 95% CI: 1.06–1.90, *P*=0.020). Compared with GG genotype, AA genotype carriers increased the risk of gastric cancer by 86% (OR = 1.86, 95% CI: 1.16–2.99, *P*=0.010). In the dominant model, GA+AA carriers had a 50% increased risk of gastric cancer compared with GG (OR = 1.50, 95% CI: 1.14–1.98, *P*=0.004). In the recessive model, the risk of gastric cancer increased by 60% compared with GG+GA (OR = 1.60, 95% CI: 1.02–2.52, *P*=0.043). The frequencies of alleles G and A in the case group were 66.24 and 33.76%, which were 72.10 and 27.90% in the control group, respectively. The results showed that the risk of developing the A allele was increased by 41% compared with the G allele (OR = 1.41, 95% CI: 1.14–1.74, *P*=0.002).

In the case group, rs10132552 TT type accounted for 50.42%, CT type accounted for 43.67%, CC type accounted for 5.91%, and in the control group accounted for 51.20, 40.33, and 8.47%, respectively; the difference was not statistically significant (*P*>0.05), showing no correlation between rs10132552 and the risk of gastric cancer (*P*>0.05). Further stratified analysis of age and gender showed that the relationship between MEG3 rs7158663 and rs10132552 and gastric cancer susceptibility is not affected by age and gender (Supplementary Tables S1 and S2). Therefore, *MEG3* rs7158663GA, AA genotype, GA+AA genotype, and A allele were associated with increased risk of gastric cancer (Table 3).

**Table 3:** Relationship between MEG3 SNPs and risk of gastric cancer

SNPs	Cases, *n* (%)	Controls, *n* (%)	OR (95% CI)^1^	*P*-value^1^
MEG3 rs7158663				
GG	215 (45.36)	290 (53.41)	1.00 (reference)	
GA	198 (41.77)	203 (37.38)	1.42 (1.06, 1.90)	**0.020**
AA	61 (12.87)	50 (9.21)	1.86 (1.16, 2.99)	**0.010**
Dominant				
GG	215 (45.36)	290 (53.41)	1.00 (reference)	
GA+AA	259 (54.64)	253 (46.59)	1.50 (1.14, 1.98)	**0.004**
Recessive				
GG+GA	413 (87.13)	493 (90.79)	1.00 (reference)	
AA	61 (12.87)	50 (9.21)	1.60 (1.02, 2.52)	**0.043**
Allele				
G	628 (66.24)	783 (72.10)	1.00 (reference)	
A	320 (33.76)	303 (27.90)	1.41 (1.14, 1.74)	**0.002**
MEG3 rs10132552				
TT	239 (50.42)	278 (51.20)	1.00 (reference)	
CT	207 (43.67)	219 (40.33)	1.08 (0.81, 1.44)	0.608
CC	28 (5.91)	46 (8.47)	0.63 (0.37, 1.09)	0.100
Dominant				
TT	239 (50.42)	278 (51.20)	1.00 (reference)	
CT+CC	235 (49.58)	265 (48.80)	1.00 (0.76, 1.31)	0.977
Recessive				
TT+CT	446 (94.10)	497 (91.53)	1.00 (reference)	
CC	28 (5.91)	46 (8.47)	0.61 (0.36, 1.04)	0.068
Allele				
T	685 (72.26)	775 (71.36)	1.00 (reference)	
C	263 (27.74)	311 (28.64)	0.92 (0.74, 1.14)	0.439

1 Adjusted by age, alcohol consumption, tea drinking, preserved food eating, and family history of cancer. Note: The bold values in the table mean that the difference in the distribution of research factors between the case group and the control group is statistically significant.

The false-positive report probability (FPRP) for positive findings at different prior probability levels are shown in Table 4. We preset 0.5 as the FPRP threshold. At the prior probability of 0.1, all the significant findings for MEG3 rs7158663 polymorphisms in different genetic models remained noteworthy. The FPRP scores of the relationship between MEG3 rs7158663 SNPs and gastric cancer susceptibility in different genetic models were all lower than the preset cut-off value of 0.5. Therefore, it can be concluded that this SNP may have a true association with gastric cancer, which is worthy of further research and verification.

**Table 4:** Probability value of false positive report on the correlation between gastric cancer risk and MEG3 rs7158663 polymorphism in the high-incidence area of gastric cancer in China

Genotype	Adjusted OR (95% CI)	*P*-value	Statistical power	Prior probability				
				0.25	0.1	0.01	0.001	0.0001
MEG3 rs7158663								
AG VS. GG	1.42 (1.06, 1.90)	0.020	0.826	**0.001**	**0.004**	**0.046**	0.329	0.831
AA VS. GG	1.86 (1.16, 2.99)	0.010	0.815	**0.001**	**0.002**	**0.019**	0.160	0.656
AG+AA VS. GG	1.50 (1.14, 1.98)	0.004	0.822	**0.000**	**0.001**	**0.012**	0.107	0.546
AA VS. GG+GA	1.60 (1.02, 2.52)	0.043	0.461	**0.003**	**0.008**	0.085	0.483	0.903
A VS. G	1.41 (1.14, 1.74)	0.002	0.913	**0.000**	**0.001**	**0.007**	0.065	0.412

The FPRP threshold level is set to 0.5, and noteworthy results are displayed in bold.

### Establishment and evaluation of genetic–environmental risk assessment model

Multivariate logistic regression results of genetic–ERFs for gastric cancer are shown in Table 5. RS was calculated as follows: RS = β1 * GRF + β2 * ERF = 1.02 * drinking or not + (−2.57) * tea drinking or not + 1.22 * eating pickled or not +0.32* rs7158663 risk allele number. The distribution of RSs was statistically significant (*P*<0.05) between the case and control groups (Table 6).

**Table 5:** Multivariate logistic regression of genetic–ERFs for gastric cancer

Variables	β	Wald χ2	OR (95%CI)	*P*-value
Drinking (yes or no)	1.02	31.89	2.78 (1.95, 3.96)	**<0.001**
Tea drinking (yes or no)	−2.57	91.76	0.08 (0.05, 0.13)	**<0.001**
Eating pickled food (yes or no)	1.22	44.14	3.37 (2.36, 4.83)	**<0.001**
rs7158663 (GG, GA, AA)	0.32	9.20	1.38 (1.12, 1.69)	**0.002**

**Table 6:** RS distribution of the study population in case–control and diagnostic ability

	*n*	RS (X ± S)	*t* value	*P*-value	AUC
Case	474	1.49 ± 0.77	−15.70	**<0.001**	0.745
Control	543	0.47 ± 1.27			

The RS was categorized into quartiles. The results showed that as the RS increased, the risk of gastric cancer increased substantially (Table 7).

**Table 7:** Grouping of genetic–environment risk factor scoring models

	Case	Control	OR (95% CI)	*P*-value
0 (<Q25)	43 (9.07)	211(38.86)	1.00 (reference)	
1 (Q25–Q50)	112 (23.63)	142 (26.15)	3.87 (2.57, 5.84)	**<0.001**
2 (Q50–Q75)	160 (33.76)	94 (17.31)	8.35 (5.51, 12.65)	**<0.001**
3 (≥Q75)	159 (33.54)	96 (17.68)	8.13 (5.37, 12.30)	**<0.001**

Note: The bold values in the table mean that the difference in the distribution of the research factors between the case group and the control group is statistically significant.

To evaluate the diagnostic capability of the model, the area under ROC curve was performed to evaluate the diagnostic ability of the genetic–environment risk assessment model. The results showed that the area under the ROC curve was 0.745, indicating that the model has a high diagnostic value (Table 6).

## Discussion

In recent years, a considerable number of studies have found that lncRNA SNPs are closely related to the risk of tumors. Li et al. [23] discovered that lncRNA *H19* rs217727 was significantly associated with lung cancer susceptibility, and homozygous AA genotype is a risk factor for lung cancer. Qiu et al. [24] found that the *HOTAIR* rs920778 TT genotype and T allele significantly increased the susceptibility of cervical cancer in the Chinese population compared with healthy controls. Another study has reported that the interaction between *HULC* rs104127 and rs2038540 and the environment could increase the risk of hepatocellular carcinoma [25]. Thus, SNPs at certain sites of lncRNA can be used as biomarkers for assessing the genetic susceptibility of tumors. There also have been studies on the risk of lncRNA and gastric cancer. Ge et al. [26] distributed that lncRNA *PTENP1* polymorphism rs7853346 may predict the susceptibility of gastric cancer. Another study has demonstrated that lncRNA *GAS5* rs145204276 was significantly associated with a reduced risk of gastric cancer [27]. Therefore, lncRNA SNPS can be used as biomarkers for gastric cancer risk.

It is reported that *MEG3* can regulate the secondary structure of *P53*. The downstream target gene of *P53* can inhibit the growth of tumor cells and *MEG3* can inhibit the growth of tumor cells by regulating the aggregation and activation of *P53* protein [28]. It may be because the *MEG3* gene polymorphism changes the gene expression level, affecting the proliferation, invasion and migration of tumor cells, which contributes to tumorigenesis. However, there were few studies on the relationship between *MEG3* polymorphism and the risk of gastric cancer. Our research first found that *MEG3* rs7158663 GA + AA carriers could significantly increase the risk of gastric cancer, and A was a risk allele of gastric cancer. A Chinese case–control study showed that the *MEG3* rs7158663 AA genotype significantly increased colorectal cancer risk compared with the GG genotype. Our findings are consistent with the above research to some extent [29]. At present, there are several studies on *MEG3* rs10132552. Wang et al. [30] showed that the risk of 3–4 grade anemia in *MEG3* rs10132552 CC genotype was significantly increased. In a study evaluating the efficacy of neoadjuvant chemotherapy for breast cancer, rs10132552 TC + CC was significantly associated with good disease-free survival [19]. In another study, *MEG3* rs10132552 was reported to be associated with treatment response in cancer patients [20]. However, there is no research on *MEG3* rs10132552 and the risk of gastric cancer. Our study was the first to focus on the risk of *MEG3* rs10132552 and gastric cancer, while we found no association between them.

The relationship between ERFs and the incidence of gastric cancer has been reported. Many meta-analysis, large cohort studies, and experimental studies suggest that chronic alcohol consumption increases the risk of gastric cancer [31]. A study conducted in North China showed that alcohol consumption could increase the risk of gastric sinus cancer by 1.765-times [32]. As one of the important ERFs for the development of gastric cancer, nitrosamines are abundant in preserved food. Our study found that drinking and preserved food eating were risk factors for gastric cancer. While tea drinking was a protective factor for gastric cancer. Lin et al. [33] found that eating salted meat and preserved vegetables were positively correlated with gastric cancer. Which was consistent with another study: the consumption of salted and preserved food could increase the burden of stomach cancer [34]. Some studies have shown that green tea drinking had a certain preventive effect on reducing the risk of gastric cancer [35,36]. In short, our conclusion is consistent with the above findings.

However, gastric cancer is the result of a combination of genetic and environmental factors. Previous studies have shown that drinking and preserved food eating were the main ERFs for gastric cancer [32,33]. At the same time, genetic factors played a key role in the occurrence of gastric cancer. In preceding studies, the use of ERFs alone often did not predict the risk of disease well, or even underestimated the risk of disease. Jeon et al. [37] showed that the area under the ROC curve for the colorectal cancer screening model based only on family history is 0.53, whereas for models based on environmental GRFs is 0.63. The genetic–environmental risk assessment model for gastric cancer established in our study comprehensively considered the effects of genetic factors and ERFs. The results showed that the model had a higher diagnostic ability for gastric cancer.

The advantage of our study lied in the large sample size, not only limited to genetic factors, but also established a model combined with environmental factors to better assess the risk of gastric cancer. However, there are certain defects in that we only consider two sites of one gene, and cannot comprehensively study the combined effects of multiple SNPS of the *MEG3* gene on the risk of gastric cancer, which will be further explored in subsequent research.

In summary, our study found that the *MEG3* rs7158663 A allele could significantly increase the risk of gastric cancer and was a risk allele of gastric cancer. In addition, drinking, tea drinking, and pickled food eating were risk factors for gastric cancer. The genetic–environmental risk assessment model established by the logistic regression model to include *MEG3* rs7158663, drinking, tea drinking, and preserved food eating has high diagnostic value, which can provide reference for the pathogenesis and diagnosis of gastric cancer in people with high incidence of gastric cancer in China.

## Conclusion

In conclusion, the present study established a geneticenvironmental risk assessment model, and is of great significance for the pathogenesis and diagnosis of gastric cancer in people with high incidence of gastric cancer in China.

## Supplementary Material

Supplementary Tables S1-S2

## Data Availability

The genotype data used to support the findings of the present study have been deposited in the Figshare repository (DOI: https://figshare.com/s/852d04268df9263fe6ec).

## Abbreviations

ERF: environmental risk factor
FPRP: false-positive report probability
GRF: genetic risk factor
lncRNA: long noncoding RNA
*MEG3*: materally expressed gene 3
OR: odds ratio
ROC: receiver operating characteristic
RS: risk score
SNP: single nucleotide polymorphism
95% CI: 95% confidence interval
