# Supplementary material for: Relationship between MEG3 gene polymorphism and risk of gastric cancer in Chinese population with high incidence of gastric cancer
Source: Biosci Rep. 2020 Nov 24;40(11):BSR20200305. doi: 10.1042/BSR20200305 (PMC7685008; doi:10.1042/BSR20200305)
Supplement: Supplementary Tables S1-S2 [file BSR-2020-0305_supp.pdf]

Supplementary Table 1 Stratified analysis of the correlation between MEG3 SNPs and gastric cancer risk by age

Model	Age≤58,n (%)		OR_{adj}(95% CI)	P_{adj}	Age>58,n (%)		OR_{adj}(95% CI)	P_{adj}
	Cases	Controls			Cases	Controls		
MEG3 rs7158663								
GG	102(47.7)	170(55.0)	1.000(reference)		113(43.5)	120(51.3)	1.000(reference)	
GA+AA	112(52.3)	139(45.0)	1.475(1.002,2.170)	**0.049**	147(56.5)	114(48.7)	1.545(1.036,2.304)	**0.033**
MEG3 rs10132552								
TT	105(49.1)	148(47.9)			134(51.5)	130(55.6)		
CT+CC	109(50.9)	161(52.1)	0.913(0.622,1.340)	0.641	126(48.5)	104(44.4)	1.100(0.740,1.636)	0.636

Adjusted by alcohol consumption, tea drinking, preserved food eating and family history of cancer.

Supplementary Table 2 Stratified analysis of the correlation between MEG3 SNPs and gastric cancer risk by sex

Model	Male,n (%)		OR_{adj}(95% CI)	*P*	Female,n (%)		OR_{adj}(95% CI)	P_{adj}
	Cases	Controls			Cases	Controls		
MEG3 rs7158663								
GG	124(46.6)	152(53.7)	1.000(reference)		91(43.8)	138(53.1)	1.000(reference)	
GA+AA	142(53.4)	131(46.3)	1.563(1.053,2.321)	**0.027**	117(56.3)	122(46.9)	1.583(1.053,2.381)	**0.027**
MEG3 rs10132552								
TT	138(51.9)	148(52.3)			101(48.6)	130(50.0)		
CT+CC	128(48.1)	135(47.7.)	1.022(0.690,1.512)	0.915	107(51.4)	130(50.0)	0.963(0.643,1.443)	0.856

Adjusted by age, alcohol consumption, tea drinking, preserved food eating and family history of cancer.
